# A Case of Sporadic Blau Syndrome with an Uncommon Clinical Course

**DOI:** 10.1155/2018/6292308

**Published:** 2018-12-30

**Authors:** Miyoko Imayoshi, Yoshiyasu Ogata, Shuichi Yamamoto

**Affiliations:** ^1^Department of Pediatrics, National Hospital Organization Higashi-Saga Hospital, 7324 Harakoga, Miyaki-machi, Saga 849-0101, Japan; ^2^Department of Pediatrics, Faculty of Medicine, Saga University, 5-1-1 Nabeshima, Saga 849-8501, Japan

## Abstract

**Background:**

Sporadic Blau syndrome (SBS), a rare systemic inflammatory disease in children, is associated with *NOD2* gene mutations. SBS is often misdiagnosed as juvenile idiopathic arthritis (JIA) because of their similar clinical manifestations. Herein, we present a case of SBS with an uncommon clinical course.

**Case Presentation:**

An 11-year-old girl with recurrent right ankle swelling for 4 years was referred to our hospital. One month before admission, she developed an intermittent high fever. She was diagnosed with systemic-onset JIA on the basis of physical and blood examination results. She was treated with ibuprofen, prednisolone, and methotrexate for 5 years. During this period, her joint lesion showed neither bone destruction nor joint space narrowing on radiography, which are characteristics of JIA. Twelve months after the termination of methotrexate treatment, she presented with bilateral panuveitis. A missense mutation, p.(R587C), was detected in her *NOD2* gene, and she was diagnosed with SBS. Then, infliximab treatment was started, and her visual acuity recovered.

**Conclusion:**

SBS may sometimes be misdiagnosed as JIA. A joint lesion without bone destruction might be a key feature to distinguish SBS from JIA. Analysis of the *NOD2* gene is recommended in such cases.

## 1. Introduction

Sarcoidosis is a chronic, multisystem, granulomatous disorder of unknown cause that rarely occurs in children [1]. Early-onset sarcoidosis (EOS) is distinctly different from adult-type sarcoidosis; it occurs in children less than 4 years of age and is characterized by the triad of skin, joint, and eye manifestations without any pulmonary lesions [1, 2]. EOS and Blau syndrome, a familial systemic inflammatory disease caused by mutations in the nucleotide-binding oligomerization domain-containing 2 (NOD2) gene. share a phenotype featuring these symptoms [3, 4]. These 2 diseases have a common genetic etiology, and EOS is now considered to be a sporadic form of Blau syndrome (sporadic Blau syndrome (SBS)) [3, 5]. A long-term follow-up study in children with EOS(SBS)/Blau syndrome showed severe complications, such as blindness, growth retardation, heart involvement, renal failure, and death [6]. EOS(SBS) may be overlooked and is sometimes misdiagnosed because of its rarity and its similarities with juvenile idiopathic arthritis (JIA) [2, 7]. Early and correct diagnosis is essential in light of the severe prognosis of the disease, specifically in the eyes [6]. The product of the *NOD2* gene, also known as the caspase activation and recruitment domain (CARD) 15, activates NF-*κ*B after recognizing a signal from a bacterial cell wall component in the leukocyte cytosol [3, 5]. Mutations in the *NOD2* gene lead to abnormally enhanced NF-*κ*B activity, which results in the occurrence of EOS(SBS)/Blau syndrome [3, 5]. Although most reported cases of EOS(SBS)/Blau syndrome involve mutations in the *NOD2* gene [5], several reports have described cases in which the characteristic clinical features of EOS(SBS) were not fully present [8–10]. In such cases, it becomes more difficult to make a correct diagnosis.

Here, we report the case of an 11-year-old girl with SBS initially diagnosed with systemic-onset JIA (sJIA) and treated for 6 years, in whom ocular involvement occurred after methotrexate termination. The diagnosis of SBS was established based on the detection of a *NOD2* gene mutation.

## 2. Case Presentation

**Figure 1:**
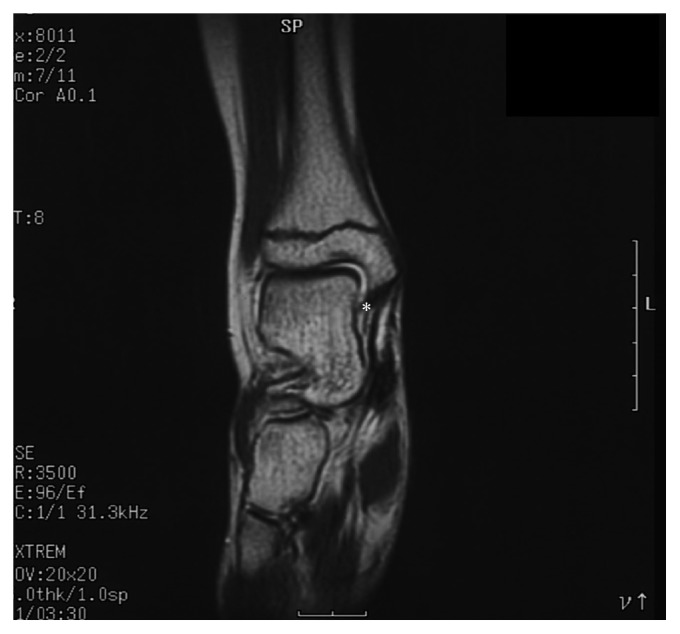
Magnetic resonance (T2-weighted) imaging of the right ankle in the patient at the age of 11 years. A small amount of fluid collection in the joint space (∗) was observed; however, there was no evidence of synovitis or destructive changes in the bone.

**Figure 2:**
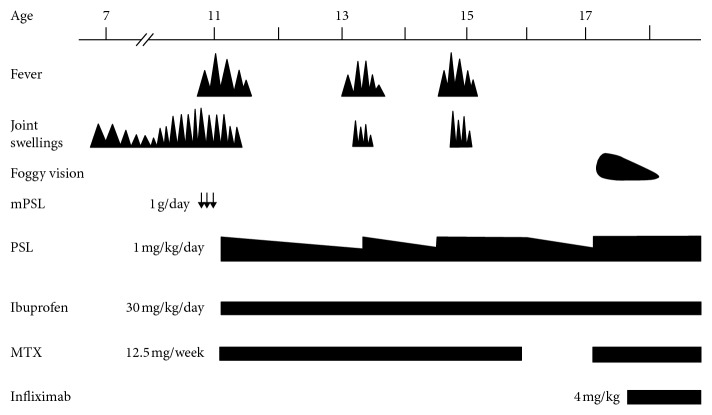
Clinical course of the patient. The patient's fever and joint swelling resolved with prednisolone, methotrexate, and ibuprofen therapy. Twelve months after cessation of methotrexate, she presented with panuveitis. She was then diagnosed with SBS. Infliximab was effective for her ocular lesion, and the dosage of prednisolone was successfully reduced. The arrows denote bolus methylprednisolone therapy. mPSL: methylprednisolone, PSL: prednisolone, MTX: methotrexate.

An 11-year-old girl was referred to our hospital. She had been suffering from recurrent right ankle swelling since she was 7 years old. She had no pain in her right ankle, and it showed no limitation of movement. She routinely underwent puncture and drainage of the joint when it swelled up. One month before admission, she had an intermittent high fever with joint swelling in her bilateral knees and right ankle. Magnetic resonance imaging revealed a small amount of fluid collection in the joint space with no evidence of synovitis (Figure 1). Although she started aspirin, the intermittent fever continued. On admission, she showed joint swelling of the right ankle but no skin rash. No eye involvement was detected upon examination by an ophthalmologist. Blood examination revealed a normal white blood cell count of 9,000/*μ*L (normal: 3,800–10,100/*μ*L) and elevated C-reactive protein level of 15.65 mg/dL (normal: less than 0.2 mg/dL). The serum immunoglobulin G level was also elevated to 2,569 mg/dL (normal: 870–1,700 mg/dL). Antinuclear antibody was borderline. Autoantibodies, including anti-dsDNA and anti-cyclic citrullinated peptide, were negative. Rheumatoid factor was negative. Blood chemistry was unremarkable. Urine tests were normal. Radiographic examination showed no hilar lymphadenopathy and no bone destructive changes in her right ankle despite a history of recurrent swelling. There was no family history of autoimmune diseases, including rheumatoid arthritis. These results led us to diagnose her with sJIA. Bolus methylprednisolone (1 g/day for 3 days), followed by prednisolone (1 mg/kg/day), ibuprofen (30 mg/kg/day), and methotrexate (15 mg/m^2^/week), was started, and her fever subsided. We attempted to reduce the dosage of prednisolone several times; however, she began to experience swelling of several joints, including the right ankle, along with a high fever. Her sJIA seemed to be corticosteroid-dependent at that time. When she was 16 years old, we discontinued methotrexate to avoid its adverse effects on the lungs. Twelve months after its cessation, she complained of “foggy” vision. Ophthalmologic examination revealed bilateral panuveitis. The findings included mutton-fat keratic precipitates and trabecular nodules in the anterior ocular segment. In addition, chorioretinal atrophy mimicking laser photocoagulation scars, retinal periphlebitis, and snowball-like vitreous opacity was observed in the posterior ocular segment. The most common form of uveitis seen in association with JIA is anterior uveitis [11]. As the granulomatous lesions spread to both her eyes, she was suspected to have sarcoidosis. However, no hilar lymphadenopathy was observed on repeated chest radiography. The serum concentration of angiotensin-converting enzyme was 9.2 U/L (normal: 8.3–21.4 U/L) and remained within the normal range. We then suspected that she had EOS(SBS). The blood sample was sent to check for a mutation of the *NOD2* gene. A missense mutation at p.(R587C) in a central nucleotide-binding and oligomerization domain, also known as a NACHT domain, of the gene was identified. Although we could not perform genetic analyses on her family members, as they had never presented with lesions of the skin, joints, or eyes, the mutation of p.(R587C) that she carried was considered to be a de novo *NOD2* mutation. The patient was finally diagnosed with a sporadic case of Blau syndrome. Methotrexate was restarted, and the dosage of prednisolone was increased soon after her panuveitis was diagnosed; however, because the prognosis of eye lesions in EOS(SBS)/Blau syndrome is reportedly poor, she was additionally treated with infliximab (4 mg/kg). Infliximab was extremely effective, and her visual acuity recovered, although partial adhesion of the iris remained. Subsequently, the dosage of prednisolone was successfully reduced to 4 mg/day, and she has remained stable without a uveitis flare. A schematic representation of her disease course is shown in Figure 2.

## 3. Discussion

Regarding the diagnosis in the present case, SBS seemed to be appropriate rather than EOS; there was no evidence of epithelioid granuloma, with the exception of the ophthalmologic findings. Because we misdiagnosed the present patient with sJIA and she had never shown symptoms of synovitis or skin lesion, we could not perform a histopathological examination. In such cases with an uncommon clinical course, the importance of genetic analysis is greatly increased for diagnosing SBS.

Nearly a period of 10 years was needed to reach a correct diagnosis. The reasons for the difficulty in the diagnosis of this patient include delayed onset of her disease, lack of skin lesions, and prolonged onset of ocular complications. In a study by Okafuji et al., it was reported that the age at disease onset was 5 years or older in only 2 of 20 EOS(SBS)/Blau syndrome patients [12]. The median ages at rash and uveitis onset were 24 months and 4.5 years, respectively [13]. Twenty-nine of 30 patients had skin rash [13], and ocular lesions were seen in 18 of 20 patients with EOS(SBS)/Blau syndrome [12]. In contrast, Arostegui et al. demonstrated that 7 of 12 patients with EOS(SBS)/Blau syndrome presented with fewer than 3 characteristics in the “classic” triad of this disease [14]. Rosé et al. showed that 29% of patients with *NOD2* mutations presented with at least 1 manifestation that can be considered atypical, either at the onset or during the course of the disease [15]. Fever may be a frequent clinical manifestation besides the triad of skin, joint, and eye lesions [12, 15]. In addition, the involvement of organs was also observed in several cases [12, 15]. Thus, clinical symptoms other than the triad should also be considered when diagnosing EOS(SBS)/Blau syndrome.

The joint involvement in this patient had several notable features. Joint swelling was limited to the right ankle at the disease onset. Most patients with EOS(SBS)/Blau syndrome present with bilateral joint lesions or polyarthritis at the disease onset [1, 12, 15]. Although we misdiagnosed this patient with sJIA upon her initial admission, she also presented with atypical joint features for JIA. Her right ankle joint was slightly swollen and warm; however, pain was minimal, and the range of motion was not substantially impaired. Neither bone destructive change on radiography nor synovitis on magnetic resonance imaging was observed (Figure 1). These observations are usually atypical in JIA [16] and might be more compatible with those observed in EOS(SBS) [17]. Although joint destruction and deformity can develop in later phases of the disease [4, 18, 19], the clinical findings observed in this case might be the characteristic features of joint lesions in early stage EOS(SBS) [12, 19]. We should have been more attentive to the manifestations of her joint lesion and suspicious of whether they fit with the clinical picture of JIA.

It is of interest that methotrexate might have prevented the onset of uveitis in this case. Methotrexate has been reported to be an effective therapy to prevent the onset of uveitis in JIA [20, 21]. Methotrexate is also used to treat EOS(SBS)/Blau syndrome and has corticosteroid-sparing effects in some EOS(SBS)/Blau syndrome patients [17, 19, 22, 23]. However, no evidence exists showing an inhibitory effect of methotrexate on uveitis onset in EOS(SBS)/Blau syndrome. Several studies have demonstrated that methotrexate in combination with corticosteroids failed to prevent the occurrence or flare of ocular involvement [10, 24]. The onset of EOS(SBS)/Blau syndrome most often starts with joint and skin lesions, and eye symptoms usually present later [15, 25]. Because uveitis is the most relevant morbidity that might result in blindness, early diagnosis and prevention of uveitis onset are particularly important. A controlled study investigating the efficacy of methotrexate against uveitis onset in EOS(SBS)/Blau syndrome is needed in future research.

Although the pathogenesis of sarcoidosis is not yet fully understood, a complex interplay has been shown between regulatory T cells and cytokines including tumor necrosis factor (TNF)-*α*, which is associated with granuloma formation [26, 27]; thus, blockade of the TNF-*α* pathway is an effective therapeutic strategy in sarcoidosis [28, 29]. Although the mechanism of granuloma formation in patients with EOS(SBS)/Blau syndrome remains unknown, TNF-*α* antagonists have been successfully used previously, as well as in our case [22, 30, 31]. Recently, paradoxical reactions to biologic agents, especially TNF-*α* antagonists, have been reported [32, 33]. In some patients treated with TNF-*α* antagonists for rheumatoid arthritis and JIA, treatment can induce the development of sarcoidosis [34, 35]. To our knowledge, there have been no reports of EOS(SBS)/Blau syndrome patients in whom clinical symptoms were worsened by TNF-*α* antagonists; however, close surveillance of patients with EOS(SBS)/Blau syndrome using TNF-*α* antagonists is warranted in order to detect the occurrence of undesirable effects due to TNF-*α*. Our patient remains under treatment with infliximab. The dosage and duration of the treatment are emerging questions in TNF-*α* antagonist therapy for patients with EOS(SBS)/Blau syndrome.

The *NOD2* gene is composed of 4 domains, including 2 CARD domains, a NACHT domain, and a leucine-rich repeat (LRR) domain [36]. Most of the mutations in EOS(SBS)/Blau syndrome are located in the NACHT domain and result in enhanced NF-*κ*B activation [37]. Because a single heterozygous mutation results in enhanced NF-*κ*B activation, Blau syndrome is inherited as an autosomal dominant trait [3]. In contrast, mutations in the LRR domain have been shown to result in the loss of function of *NOD2*, which is one of etiologies observed in patients with Crohn's disease [38, 39]. Okafuji et al. investigated 20 patients with EOS(SBS)/Blau syndrome harboring *NOD2* mutations and reported that a link between the clinical severity of the disease and basal NF-*κ*B activity was not established [12]. However, in a comparison between the 2 most frequent mutations, p.(R334W) and p.(R334Q), visual impairments tended to be more severe in patients with p.(R334W) [12]. Rose et al. found that the p.(R334W) mutation was significantly less frequent in patients with an atypical phenotype [15]. The patient in our case had a p.(R587C) missense mutation, which has been reported in both EOS(SBS) and Blau syndrome cases [14, 15, 40]. Interestingly, in the study by Rose et al., all 3 Blau syndrome patients carrying p.(R587C) mutation presented with an extended phenotype including fever, erythema nodosum, and pericarditis [15]. However, it remains unclear whether the p.(R587C) mutation in the *NOD2* gene is related to atypical manifestations, such as those observed in the present case. Identification of additional cases of EOS(SBS)/Blau syndrome in the future will reveal how different types of mutations in the *NOD2* gene affect the clinical course and manifestations of this disease.

In conclusion, patients with EOS(SBS) do not always present with typical clinical symptoms. Because the prognosis of ocular involvement in patients with EOS(SBS) is poor, EOS(SBS) should be diagnosed promptly and correctly. Clinicians should not place undue importance on the triad of the disease and should pay more attention to joint manifestations in patients with arthritis in order to distinguish EOS(SBS) from JIA. Characteristic joint manifestations and mutations in *NOD2* may represent hallmarks in the early diagnosis of EOS(SBS). Further studies investigating the precise clinical course and manifestations in patients with EOS(SBS)/Blau syndrome are needed for a better understanding and early diagnosis of this disease.
